# Supplementary material for: Screening for Chagas Disease during Pregnancy in the United States—A Literature Review
Source: Trop Med Infect Dis. 2021 Nov 26;6(4):202. doi: 10.3390/tropicalmed6040202 (PMC8704518; doi:10.3390/tropicalmed6040202)
Supplement: Supplementary file 1 [file tropicalmed-06-00202-s001.zip › tropicalmed-1448950-supplementary.pdf]

Tropical Medicine and Infectious Disease

MDPI

Supplementary material of Screening for Chagas Disease during Pregnancy in the United States—A Literature Review

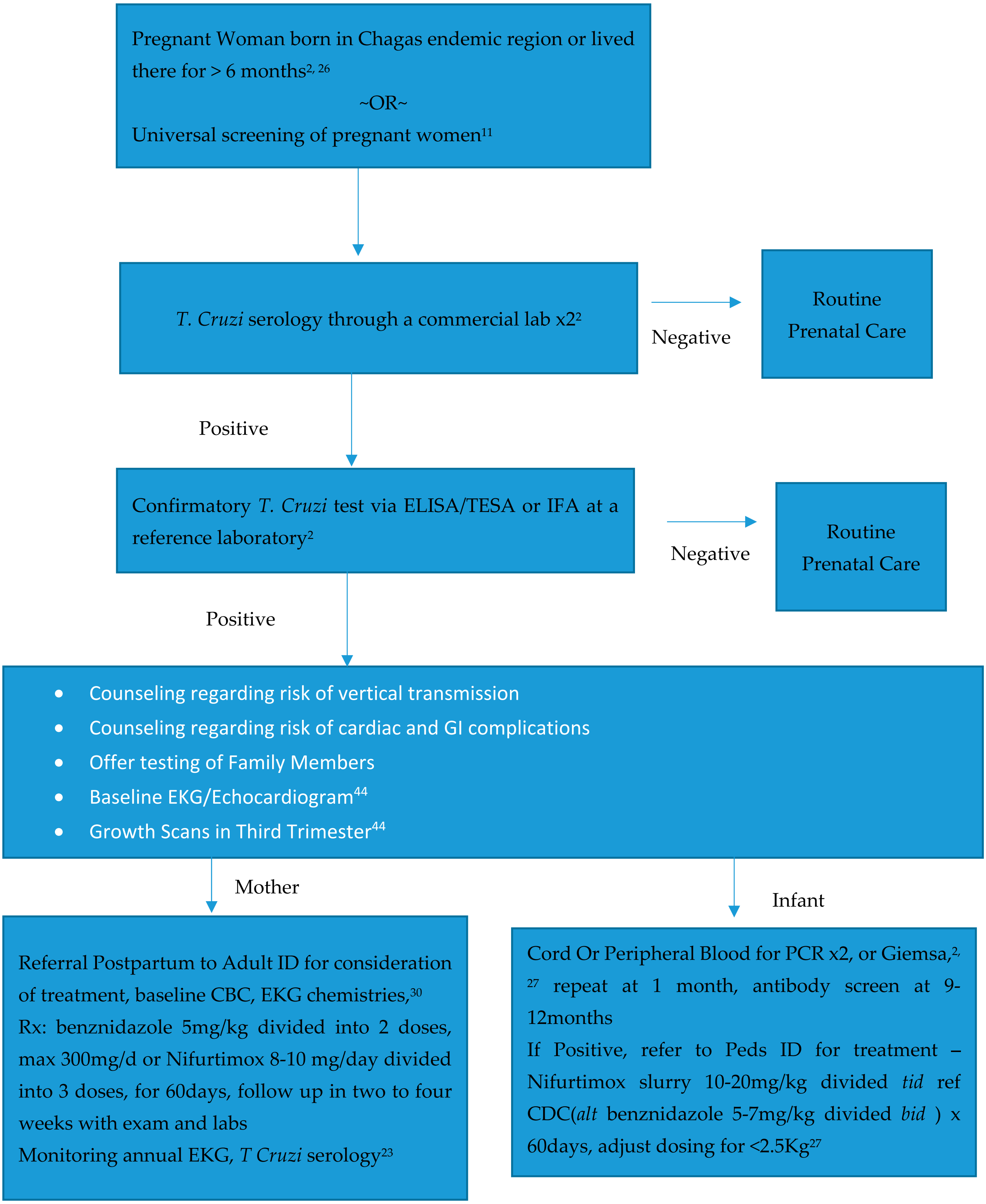

Figure S1. Proposal for Screening and Management of Chagas Disease in Pregnancy in the US.
